# Description of a new *Tegenaria* Latreille, 1804 from southern Turkey with remarks on the *Tegenaria ariadnae* species-complex (Arachnida, Araneae)

**DOI:** 10.3897/zookeys.935.52089

**Published:** 2020-05-21

**Authors:** Dragomir Dimitrov

**Affiliations:** 1 National Museum of Natural History, Bulgarian Academy of Sciences, 1 Tsar Osvoboditel Blvd, 1000 Sofia, Bulgaria National Museum of Natural History Sofia Bulgaria

**Keywords:** Cave fauna, endemic, funnel weaver spiders, new species, taxonomy

## Abstract

A new cave-dwelling species of *Tegenaria*, *T. lazarovi***sp. nov.**, is described from southern Turkey, based on both sexes. The new species belongs to the *T. ariadnae* species-complex which is distributed in the Eastern Mediterranean (Crete, northern Libya). The new species is compared to its morphologically closest congeners. New taxonomically relevant pictures are given for two of them. The distribution of the *Tegenaria ariadnae* species-complex is summarized and discussed.

## Introduction

With 112 valid species (WSC 2020), *Tegenaria* Latreille, 1804 is one of the largest genera in the spider family Agelenidae. The genus is very species-rich in Turkey. Demir and Seyyar (2017) listed 31 species, Özkütük et al. (2017) and Topçu and Demircan (2018) added two more, increasing the number of known species from the country to 33. Many of these species are known by one sex only. Especially high is the number of species where only the female is known (12 spp.), while in just one species only the male is known. The remaining 20 species are known by both sexes. The species known only by females are described mostly from caves by Brignoli (1972b, 1977, 1978a, 1978b) and the absence of the male sex is a cause of certain difficulties in the taxonomy of the genus.

While processing unidentified material collected in 2006 by my colleagues Stoyan Lazarov and Pavel Stoev in Turkish caves, I discovered an unknown *Tegenaria* species captured in an unnamed cave situated between Anamur and Silifke, southern Turkey. The species is described below and its possible relationships, as well as the distribution of the *T. ariadnae* species-complex, are discussed.

## Material and methods

The material was preserved in vials with 80% ethanol in the field. The specimens were examined and measured using a Wild M5A stereomicroscope; all measurements are in mm. Pictures were taken with a Canon EOS 1100D digital camera attached to a Carl Zeiss Amplival microscope. The drawings were executed on a Wacom tablet and using Adobe Illustrator graphic design software. The map was generated with the SimpleMappr API. Colour was described from specimens preserved in ethanol. The male palp and epigyne were dissected in order to be studied and illustrated. The epigyne was cleared in lactic acid. Leg measurements formula: total length (coxa + trochanter, femur, patella, tibia, metatarsus, tarsus). Tarsus length includes claws.

**Abbreviations:** Morphology. **ALE** – anterior lateral eyes, **AME** – anterior median eyes, **C** – conductor, **CO** – copulatory openings, **DBTA** – dorsal branch of the tibial apophysis, **DPC** – dorsal part of terminal end of the conductor, **LBTA** – lateral branch of the tibial apophysis, **MA** – median apophysis, **MPE** – median plate of epigyne, **PLE** – posterior lateral eyes, **PME** – posterior median eyes, **R** – receptacles, **VPC** – ventral part of terminal end of the conductor.

**Institutions. MBCG** – Museo Civico Scienze Naturali Enrico Caffi, Bergamo, Italy; **MCSN** – Museo Civico di Storia Naturale, Verona, Italy; **NMNHS** – National Museum of Natural History, Sofia, Bulgaria; **SMF** – Senckenberg Research Institute, Frankfurt, Germany.

## Taxonomy

### Family Agelenidae C. L. Koch, 1837

#### Genus *Tegenaria* Latreille, 1804

##### Tegenaria lazarovi sp. nov.

Taxon classificationAnimaliaAraneaeAgelenidae

38DDCA80-7CEB-56E2-91BC-8BF5B272570D

http://zoobank.org/CADBD8E5-2299-4BC4-AFF6-9CFCAA55D011

Figs 1–4 , 5–6

###### Type material.

♂ holotype, 2 ♀ paratypes, Turkey, Silifke distr., Karatepe village, unnamed cave on the left side of the road Anamur-Silifke, Akçalı Dağları Mts. 36; 36°10'55"N, 33°26'41"E, altitude 182 m, wet sand; 16.07.2006; P. Stoev and S. Lazarov leg. (NMNHS); 1 ♀ paratype, the same data as holotype (SMF).

###### Other material.

3 ♀ juveniles, the same data as holotype (NMNHS).

###### Comparative material examined.

*Tegenaria vallei* Brignoli, 1972. ♂ holotype, Libya, Cyrenaica, Lete Cave, Benghasi, 06.04.1966, Valle and Bianchi leg., 1 ♂ paratype, the same locality as holotype, 31.12.1967, Valle leg. (MBCG); 1 ♀ paratype, the same locality as holotype, 31.12.1967, Valle leg. (MCSN); *Tegenaria pieperi* Brignoli, 1979. ♀ holotype, Crete, Sitia, Agios Georgios, Megalo Katafigi Cave, 21.05.1977, H. Pieper leg. (MCSN).

###### Etymology.

The species is dedicated to my colleague, Bulgarian arachnologist Stoyan Lazarov who provided me with the type material. He was chosen over Pavel Stoev by tossing a coin.

###### Diagnosis.

**Figures 1–4.**
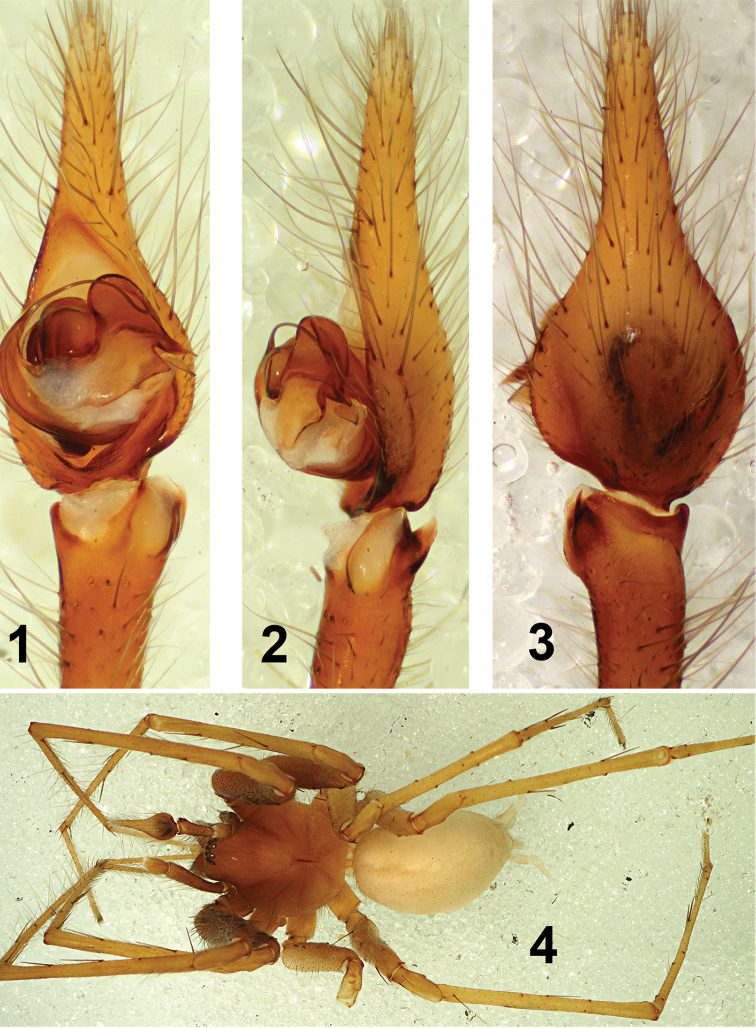
*Tegenaria lazarovi* sp. nov. male holotype. Palp ventral (**1**), palp retrolateral (**2**), palp dorsal (**3**), habitus (**4**).

**Figures 5–7.**
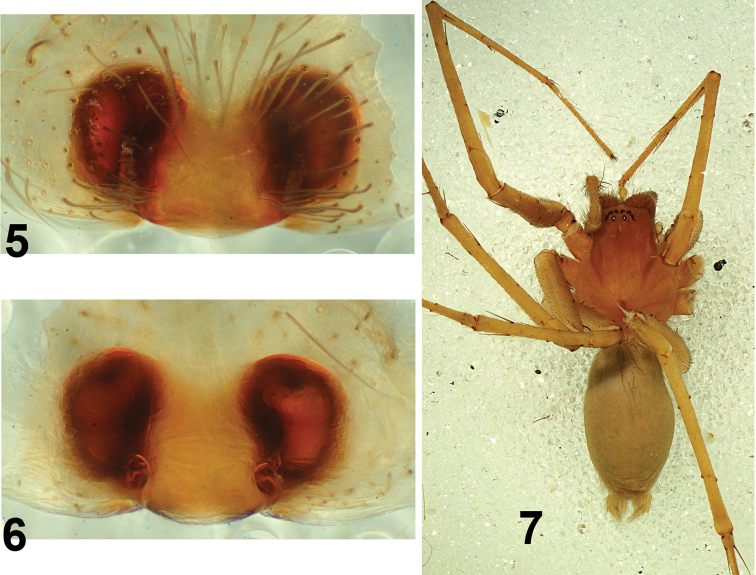
*Tegenaria lazarovi* sp. nov. female paratype. Epigyne ventral (**5**), vulva dorsal (**6**), habitus (**7**).

**Figures 8–12.**
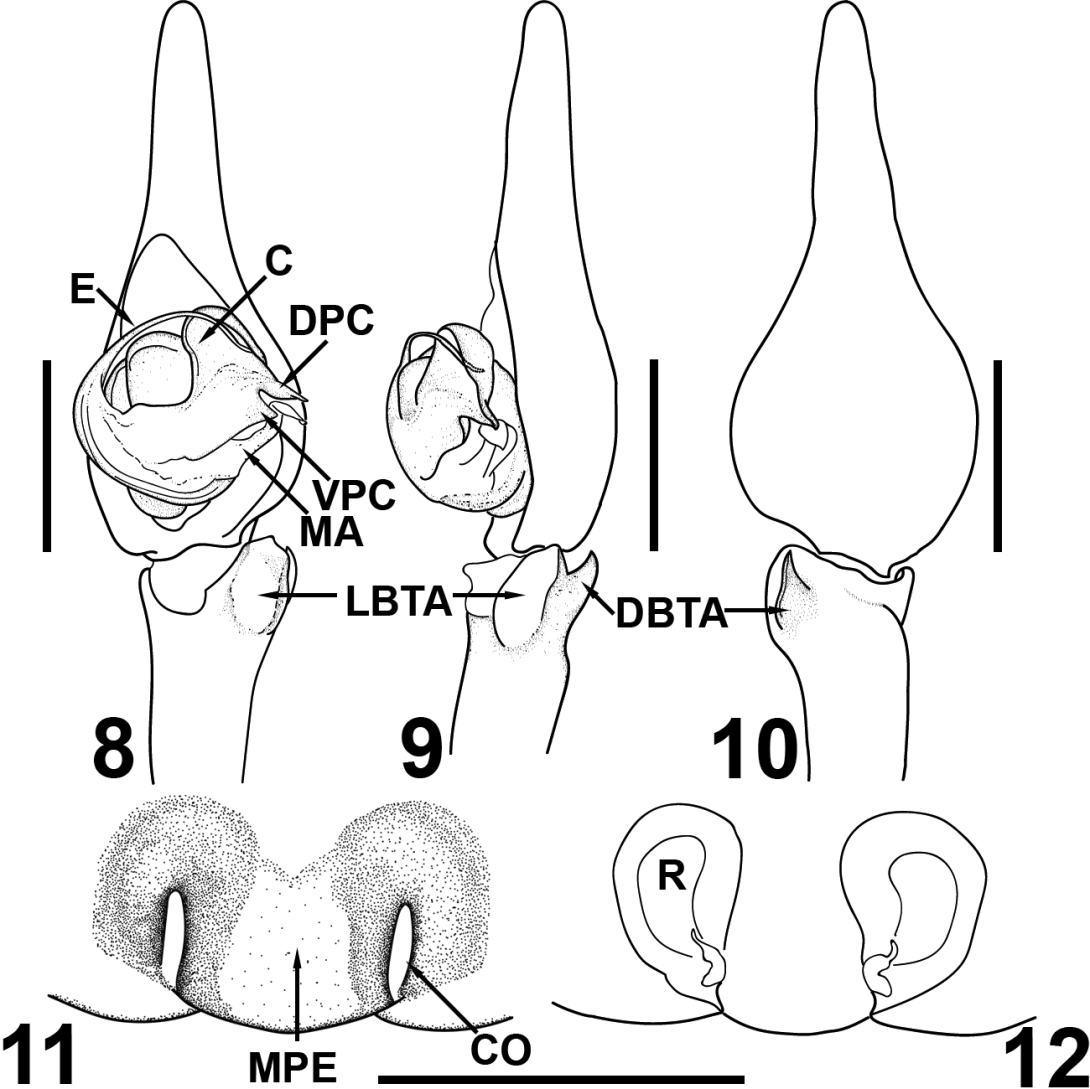
*Tegenaria lazarovi* sp. nov. Male holotype (**8–10**). Palp ventral (**8**), palp retrolateral (**9**), palp dorsal (**10**). Female paratype (**11, 12**). Epigyne ventral (**11**), vulva dorsal (**12**) Scale bars: 1.0 mm.

The new species fits well in the genus *Tegenaria* as defined by Bolzern, Burckhardt and Hänggi (2013) according to its straight trochanters, the absence of dorsal spines on the patellae and the shape of the conductor. It appears closest to *Tegenaria ariadnae* Brignoli, 1984. The males can be separated by the following characters:(1) The DBTA of *Tegenaria lazarovi* sp. nov. is claw-shaped, with a sharp tip (Figs 2, 3, 9, 10), while in *T. ariadnae* it is more massive and with a blunt tip (Bolzern, Burckhardt and Hänggi 2013: 769, fig 14R); (2) A lighter, less sclerotized LBTA (Figs 1, 2, 8, 9), more sclerotized in *T. ariadnae* (Bolzern, Burckhardt and Hänggi 2013: 769, fig 14R, 16M); (3) A pointed, triangular VPC (Figs 1, 8), trapezoid in *T. ariadnae* (Bolzern, Burckhardt and Hänggi 2013:769, fig 14Q); the females of the two species can be separated by (4) the trapezoid epigynal median plate (MPE) with a broader distal part in *Tegenaria lazarovi* sp. nov. (Figs 5, 11), which in *T. ariadnae* is broader in the basal part (Bolzern, Burckhardt and Hänggi 2013:769, fig 14S); (5) The copulatory openings are perpendicular to the MPE and positioned to its distal part (Figs 5, 11) while in *T. ariadnae* they are horizontal, positioned much higher (Bolzern, Burckhardt and Hänggi 2013: 769, fig 14S); (6) The receptacles are larger, kidney-shaped (Figs 6, 12) while being smaller and more oval in *T. ariadnae* (Bolzern, Burckhardt and Hänggi 2013: 769, fig 14T, 16D).

###### Description.

**Male.** Measurements. Total length (including spinnerets) 7.66; carapace length 3.23, width 2.50; chelicerae length 1.43; clypaeus height 0.22; eye diameters AME 0.075, ALE 0.090, PME 0.090, PLE 0.090; AE separated from each other by 0.020, ALE almost touching PLE, PME separated from each other by 0.14 and from PLE by 0.080; abdomen length 4.43 (including spinnerets), width 2.05; Leg measurements I 20.71 (1.65, 5.55, 1.13, 4.80, 4.80, 2.40), II 16.96 (1.50, 4.13, 1.25, 3.75, 4.15, 2.18), III 15.79 (1.45, 3.75, 1.13, 3.45, 3.90, 2.10); IV 22.55 (1.58, 4.88, 1.13, 4.60, 5.40, 2.48). Leg spination typical for the genus. Coloration (Fig. 4). Carapace light brown to yellow, darker in the anterior half, gradually lightening posteriorly. Chelicerae light brown. Legs I, II light brown, legs III, IV yellow. Sternum without pattern, yellow in the center, gradually darkening to light brown at the edges. Palpal femur light brown, other segments gradually lightening, yellowish. Abdomen white, without pattern. Other somatic characters. Chelicerae with 2–3 promarginal and 5 retromarginal teeth. All trochanters straight. Colulus is a single trapezoid plate, slightly notched in the middle of the distal part. Palp (Figs 1–3, 8–10). Femur length 1.80; patella length 0.60; tibia length 2.03; cymbium length 2.93. Tibia with short retrolateral apophysis with dorsal and lateral branches. Dorsal branch (DBTA) claw-shaped with sharp end. Lateral one (LBTA) rounded, less chitinized, whitish, surrounded by a light brown more sclerotized strip (Figs 2, 3, 9, 10). Cymbium long and narrow with a slight depression dorsally (Figs 2, 9). Embolus comparatively long and thin, originating at 9 o’clock and ending at 2 o’clock position. Conductor short and broad, distal portion rounded, both dorsal and ventral part of terminal end sharp. MA membranous, long and narrow, ending in a more chitinized plate, situated between the dorsal and ventral part of the conductor’s terminal end (Figs 1, 8).

**Female.** Measurements. Total length (including spinnerets) 8.30; carapace length 3.80, width 2.50; chelicerae length 0.88; clypaeus height 0.29; eye diameters and arrangement as in male; abdomen length 4.50 (including spinnerets) width 2.25; Leg measurements I 19.14 (1.58, 4.50, 1.13, 5.63, 4.50, 1.80), II 16.71 (1.58, 4.05, 1.13, 3.90, 3.90, 2.15), III 15.41 (1.50, 3.60, 1.13, 3.38, 3.90, 1.90); IV 22.81 (1.73, 4.80, 1.13, 4.65, 5.25, 2.25). Leg spination typical for the genus. Female palpal tibia with 2 dorsal and 2 prolateral spines. Coloration (Fig. 7). Carapace, chelicerae and sternum as in male. All legs yellow. Palpal femur, patella and tibia yellow, tarsus light brown. Abdomen whitish to light gray, darker than in male, without pattern. Median plate of epigyne light brown, framed laterally by dark brown spots. Other somatic characters. Chelicerae with 3 promarginal and 5 retromarginal teeth. All trochanters straight. Colulus is a single trapezoid plate, slightly notched in the middle of the distal part. Epigyne and vulva (Figs 5, 6, 11, 12). Width 0.98. Epigynal median plate trapezoid, with M-shaped base, broader in the distal part. Copulatory openings vertical, situated on both sides of the median plate (Figs 5, 11). Posterior sclerite absent. Receptacles large and oval (Figs 6, 12).

**Figures 13–18.**
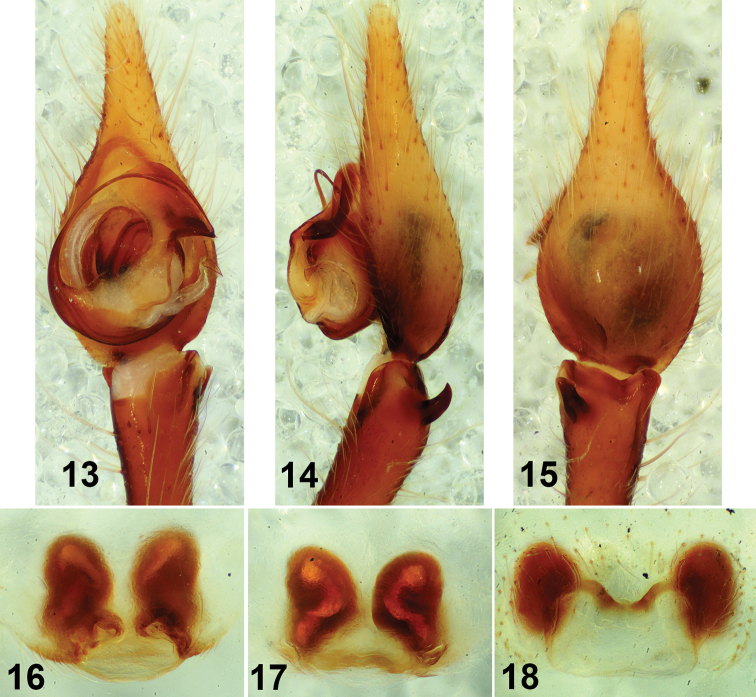
*Tegenaria vallei* Brignoli, 1972 (**13–17**) Male holotype (**13–15**) palp ventral (**13**) palp retrolateral (**14**) palp dorsal (**15**) Female paratype (**16, 17**) epigyne ventral (**16**) vulva dorsal (**17**) *Tegenaria pieperi* Brignoli, 1979 female holotype, epigyne ventral (**18**).

###### Distribution.

Known only from the type locality in southern Turkey.

###### Remarks.

**Figure 19.**
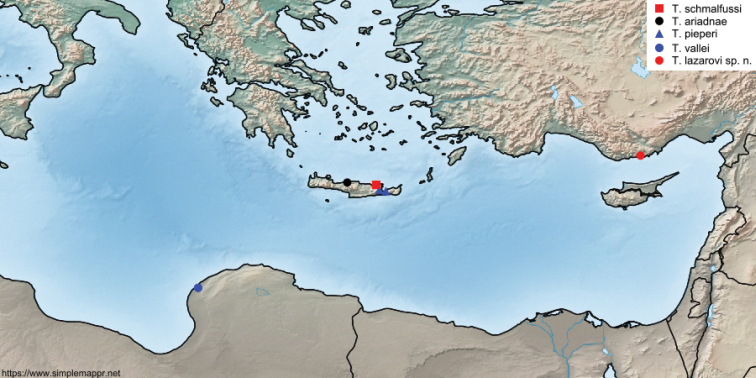
Distribution of the *Tegenaria ariadnae* species-complex.

Two *Tegenaria* species known from Crete, namely *Tegenaria pieperi* Brignoli, 1979 and *Tegenaria schmalfussi* Brignoli,1976 are also similar to *T. ariadnae* and *T. lazarovi* sp. nov. *Tegenaria pieperi* Brignoli, 1979 is known only by the female which differs from *T. lazarovi* sp. nov. by the rectangular MPE and the smaller and much higher positioned receptacles (Fig. 18). The male of *T. schmalfussi* differs from *T. lazarovi* sp. nov. by the lack of VPC and the smaller DBTA and LBTA (Bosmans et al. 2013, figs 50–52); the female can be distinguished by the smaller MPE and different shape of the receptacles (Bosmans et al. 2013, figs 53–54). I would include in this species complex also *Tegenaria vallei* Brignoli, 1972, known from a cave near Benghazi, Libya. Its male can be distinguished by having conductor with entirely missing VPC (Fig. 13) and different DBTA and LBTA (Figs 14, 15). The female differs by the oval MPE (Fig. 16) and the longer receptacles (Fig. 17). The *T. ariadnae* species-complex has a typical Eastern Mediterranean distribution with three species known from Crete, one from northern Libya and one from southern Turkey (Fig. 19). It is interesting that *T. lazarovi* sp. nov. appears more closely related to species inhabiting Crete and northern Libya than to any of the *Tegenaria* species known from the Turkish mainland. However, the current knowledge of the spider fauna of the easternmost Mediterranean (especially in north-eastern Africa) is insufficient to provide an explanation for this observation.

## Supplementary Material

XML Treatment for Tegenaria lazarovi
